# Mr. Wagstaffe's Case of Inflamed Mamma

**Published:** 1801-03

**Authors:** M. F. Wagstaffe


### To the Editors of the Medical and Physical Journal.

GENTLEMEN,

ON the 25th of November I was desired to visit the child of Mrs. Browne, late in the evening, of which child I had delivered her on the 31st of the preceding month. Upon examining the child, who by its violent shrieks and writhing of the body seemed to be in excruciating pain, the appearances were as follow: A violent inflammation appeared, extending longitudinally from the upper surface of the right breast to the umbilicus; and laterally from the inferior angle of the scapula over the ribs to the inferior portion of the left breast. To trace its circumference, we will commence from the inferior angle of the right scapula passing nearly to the axilla, enveloping almost the whole of the right breast, descending to the scrobic. cordis, then ascending as high as the nipple of the left breast, then suddenly descending in a strait line to a parallel with the umbilicus, passed close upon its upper surface, declining a little on the right side, then gradually diminishing in its ascent to the inferior angle of the scapula.

Upon making inquiry about her previous health, the mother stated to me, that the infant when she retired to rest the preceding evening was in apparent good health; which it had enjoyed from the birth. In the night she was waked by its crying, and immediately applied it to the breast, which it eagerly embraced, and as speedily relinquished; it continued crying till morning, when she undressed it, to examine whether any external injury was the cause, having previously supposed it to be an affection of the bowels. On removing the cloaths she observed both breasts turgid, milk oozing from the nipples, and the right breast much inflamed; these appearances induced her to apply a common poultice, which not affording the desired relief, she sent to me. On my arrival, I found the appearances as I have just stated.

I desired linen cloths to be dipped in Aq. lytharg. acet. comp. applied to the inflamed parts, and kept constantly wet; evacuated the intestines with a little rhubarb and calomel, and gave two drops of tinct. opii every hour during the night. The following day the saturnine lotion was continued with one drop of Tinct. opii every two hours. The inflammation did not materially abate, but became circumscribed to the limits before mentioned.

On the 27th the abdomen became much distended and very tense. I directed the intestines to be again evacuated by infusum sennæ, giving a tea spoonful every quarter of an hour until til it ſhould operate freely, and the abdomen to be fomented frequently afterwards, anointing it with ung. ſaturninum.— Theſe remedies afforded the child relief for about two hours, the fomentation and ointment uniformly producing the ſame effect when repeated.

The 28th, obſerving a fluctuation under the inflamed integuments, I directed a ſoft poultice to be applied, and gave the infant ℥ſs decoct. cinch. with two drops of Tinct. opii three times a day, which it ſwallowed with ſome difficulty. The following day, Nov. 29, the abſceſs broke immediately over the ſcrobic. cordis, when not leſs than four ounces of pus were evacuated. The infant continued the medicines during this day, but on the following (Nov. 30.) declined even the breaſts, which it returned to a little the day after, but refuſed wine and every other ſupport.

The wound now put on a formidable appearance, and I fully expected the infant would have ſunk with exhauſtion. A carrot poultice was applied and repeated frequently for the three following days, when a large portion of the integuments ſloughed off, and left part of the ribs and ſternum expoſed, excepting their muſcular covering. From this time the child had more frequent recourſe to the breaſt, ſwallowed a little red wine, and took the bark as before, and has continued to improve in health ever ſince; the bark however affecting the bowels, was obliged to be omitted. I dreſſed the inner ſurface with yellow digeſtive, covering the whole with a pledget of cerat. epulot. I ſhould mention, that an abſceſs had alſo formed under the inferior angle of the ſcapula, and evacuated itſelf by the aperture over the ſternum. By turning the child with the face downwards, the integuments have adhered to the muſcles beneath, from that part to the right breaſt, the abſceſs being firſt completely evacuated.

The wound is at this time, Jan. 4, nearly cicatrized, and the child perfectly well.

I have juſt ſtated the foregoing caſe, to ſhow how much injury a child a month old is able to ſuſtain. The inflammation was undoubtedly the cauſe of the following miſchief; but to what exciting cauſe is this inflammation, ſo extenſive in its limits and ſo rapid in its progreſs, to be referred?

Is it to be attributed to the abundant ſecretion of milk in the breaſts producing diſtention?

I am, &c.

M. F. WAGSTAFFE.

